# Introduction of New Vaccines: Decision-making Process in Bangladesh

**DOI:** 10.3329/jhpn.v31i2.16385

**Published:** 2013-06

**Authors:** Jasim Uddin, Haribondhu Sarma, Tajul I. Bari, Tracey P. Koehlmoos

**Affiliations:** ^1^icddr,b, GPO Box 128, Dhaka 1000, Bangladesh;; ^2^Expanded Programme on Immunization, Directorate General of Health Services, Ministry of Health and Family Welfare, Government of the People's Republic of Bangladesh, Mohakhali, Dhaka 1212, Bangladesh

**Keywords:** Decision-making, Qualitative research, Vaccines, Bangladesh

## Abstract

The understanding of the decision-making process in the introduction of new vaccines helps establish why vaccines are adopted or not. It also contributes to building a sustainable demand for vaccines in a country. The purpose of the study was to map and analyze the formal decision-making process in relation to the introduction of new vaccines within the context of health policy and health systems and identify the ways of making decisions to introduce new vaccines in Bangladesh. During February-April 2011, a qualitative assessment was made at the national level to evaluate the decision-making process around the adoption of new vaccines in Bangladesh. The study population included: policy-level people, programme heads or associates, and key decision-makers of the Government, private sector, non-governmental organizations, and international agencies at the national level. In total, 13 key informants were purposively selected. Data were collected by interviewing key informants and reviewing documents. Data were analyzed thematically. The findings revealed that the actors from different sectors at the policy level were involved in the decision-making process in the introduction of new vaccines. They included policy-makers from the ministries of health and family welfare, finance, and local government and rural development; academicians; researchers; representatives from professional associations; development partners; and members of different committees on EPI. They contributed to the introduction of new vaccines in their own capacity. The burden of disease, research findings on vaccine-preventable diseases, political issues relating to outbreaks of certain diseases, initiatives of international and local stakeholders, pressure of development partners, the Global Alliance for Vaccines and Immunization (GAVI) support, and financial matters were the key factors in the introduction of new vaccines in Bangladesh. The slow introduction and uptake of new vaccines is a concern in the country. Rapid action on the application of GAVI support and less time taken by the Government in processing the implementation and administrative work may expedite the introduction of new vaccines in future in this country.

## INTRODUCTION

Immunization, a proven tool for controlling and eliminating life-threatening infectious diseases, can avert 2-3 million deaths each year globally. It is one of the most cost-effective health investments (1). Universal immunization of children against six vaccine-preventable diseases, such as tuberculosis, diphtheria, whooping cough (pertussis), tetanus, polio, and measles, is crucial to reducing infant and child mortality (2-7). Consequently, childhood immunization remains a key channel for the attainment of the Millennium Development Goal 4 (MDG 4) of reducing child mortality by two-thirds within 2015.

In recent years, vaccines have been available against other diseases that are also important in terms of public-health perspectives. However, most developing countries did not have the means of accessing, evaluating, and implementing these newly-developed vaccines (8). This situation led to a divergence in global vaccine-use, and many children who were in most need were deprived of access to the new vaccine options (9). Of these new vaccines, *Haemophilus influenzae* type b (Hib), hepatitis B (Hep-B), pneumococcal conjugate vaccine (PCV), and rotavirus vaccines are particularly geared toward children of developing countries where the burden of disease is high (10-12).

These new vaccines differ from those originally included in the Expanded Programme on Immunization (EPI). New vaccines tend to be considerably more expensive than existing vaccines, and some targeted diseases are relatively ‘hidden’ and, therefore, may lack demand from public and political perspectives (13). The slow introduction and uptake of new vaccines reinforces the importance of information for making decisions (12). After the new vaccines are licensed, policy-makers require information on the burden of disease, costs of vaccines, and cold-chain facilities to make decision to introduce new vaccines (14-16). The decision-making process is complex, notably because it is driven by many different factors and involves multiple actors. Increasing the understanding of the decision-making process in the introduction of new vaccines helps establish why vaccines are adopted or not and contributes to building a sustainable demand for vaccines in a country (17).

To control the vaccine-preventable diseases, the Government of Bangladesh has been working to expand the EPI by introducing new vaccines. Bangladesh introduced the pentavalent Hib vaccine in January 2009 to prevent severe child pneumonia and meningitis. However, the formal decision-making process in relation to the adoption of vaccines was never studied in Bangladesh. The purpose of the present study was, therefore, to map and analyze the formal decision-making process in the introduction of new vaccines within the context of health policy and health systems and identify the ways of making decisions to uptake new vaccines in the country.

## MATERIALS AND METHODS

During February-April 2011, a qualitative assessment was made at the national level to assess the decision-making process in relation to the adoption of new vaccines in Bangladesh.

For policy analysis of the decision-making process in the adoption of vaccines, the study included: policy-level people, programme heads or associates, and key decision-makers of the Government, private sector, non-governmental organizations (NGOs), and international agencies at the national level.

In total, 13 key informants were purposively selected from relevant government, non-government and international agencies, and most informants were the members of any or more committees on EPI.

### Collection of data

Data were collected by interviewing key informants and reviewing secondary documents.

*Key informant interviews:* Considering the complexity of the decision-making process as it is driven by many different factors and involves multiple actors, the key informants were selected from the Ministry of Health and Family Welfare (MoHFW); Ministry of Local Government, Rural Development & Co-operatives (MoLGRD); Ministry of Finance; Directorate General of Health Services (DGHS); EPI; Dhaka City Corporation; World Health Organization (WHO); United Nations Children's Fund (UNICEF); representatives from different committees, including National Committee for Immunization Practice (NCIP), Scientific and Technical Sub-committee (STSC), Inter-agency Coordination Committee (ICC), National Steering Committee for Polio Eradication and Measles Control (NSCPEMC), and NGOs. Data were collected from the actors involved in decision-making on the following: time taken in decision-making, factors that influence decision-making, decisions on financing and barriers faced in the introduction of new vaccines, steps taken to solve the problems, why vaccines are adopted or not, and demand for new vaccines.

*Review of secondary documents:* Secondary documents relating to the adoption of new vaccines were collected and reviewed. Types of documents reviewed included: comprehensive multi-year plan of the national immunization programme of Bangladesh for 2006-2010 and 2011-2016, systems research relating to EPI, policy analysis-related documents on new vaccines, immunization surveillance system-related documents, unpublished documents relating to upcoming vaccines, and minutes of meetings of different committees.

### Analysis of data

*Secondary data:* Evidence from the review of policy documents provided general information necessary to describe the general context and introduction of new vaccines.

*Analysis of qualitative data:* Analysis of qualitative data began following the initial data collection from the field and led to refinements as the study progressed. The interviewers prepared transcripts after the completion of each interview. At first, the transcripts were carefully read, the main findings were listed, and then the coding of main findings was carried out. After reading, re-reading, and coding the texts, the main themes were begun to formalize. Each theme was then examined separately and fully within the available data.

### Ethical considerations

The respondents were interviewed after obtaining their informed consents. Efforts were made to ensure that all respondents were properly informed about the study and they thoroughly understood their involvement. Participation was voluntary. The participants were ensured that refusal would have no adverse consequences for them. They were also assured that the information provided by them would be used for research purposes only and would not be shared anywhere by their names. Interviews were conducted according to the convenience of the respondents.

The Research Review Committee and Ethical Review Committee of icddr,b approved the study before its implementation.

## RESULTS

### Actors involved in decision-making

#### Government

The Government of Bangladesh is the main actor in making decisions on the introduction of new vaccines. The respondents mentioned that the key people involved in decision-making are: Director, Primary Health Care (PHC); Programme Manager, EPI; and Director General, DGHS. Their roles included the formulation of policy about the implementation of the existing EPI vaccines and taking initiatives for the introduction of new vaccines. They are also the key persons in all national-level EPI-related committees. The Joint Secretary, PHC-WHO at the MoHFW and the Joint Secretary, Budget, at the Ministry of Finance are particularly important. Although they do not participate in different forums relating to the introduction of new vaccines, their signatures in the Global Alliance for Vaccine and Immunization (GAVI) application for the introduction of new vaccines are essential.

#### Academicians

According to the key informants, the academicians also play important roles in introducing new vaccines. The academicians included professors of paediatrics, immunology, virology, and liver transplantation. Most respondents reported that high value is given to the opinions of the academicians in relation to the introduction of new vaccines. They stated that, if the specialists and academicians do not agree with the introduction of any vaccine, the concerned authority would not approve its introduction.

#### Researchers

All the informants reported that the introduction of new vaccines is determined based on scientific evidence. They stated that researchers and scientists are very important in providing scientific information on the need for the introduction of new vaccines. Most respondents mentioned the contribution of researchers in the introduction of the Hib vaccine. They said that, before the introduction of the Hib vaccine, good data were available to understand the burden of disease. According to them, a survey was conducted by the international research institutions and medical colleges. Findings of studies provided information on the burden of diseases and the need for the introduction of Hib vaccine. They shared the findings with the policy level, which influenced the introduction of the vaccine in the country. The respondents informed that the researchers conduct studies and make recommendations to determine which vaccine should be introduced on a priority basis.

#### Representatives from professional associations

Representatives from different professional associations play an important role in the introduction of new vaccines. The associations included: Bangladesh Paediatric Association, Bangladesh Medical Association (BMA), and Bangladesh Hepatological Society (BHS). Some of them are members of different committees on EPI, and they influence in introducing a new vaccine. One respondent said:

You know.…BHS has been pressurizing the Government to introduce Hep-B birth dose. At a certain point, our Hon'ble Health Minister became convinced about it, and he is in favour of introducing it at least at the facility level before its introduction at the outreach centres.

#### Media

The informants stated that the media generally do not have a major role in the introduction of new vaccines. The media play roles in the implementation of a new vaccine but their role in decision-making is minor. Normally, the vaccines are introduced in phases. The respondents mentioned that the reason for the lower level of influence of the media is that, if the media are involved in decision-making, they may publicize about the vaccine all over the country, which may create confusion among the people.

#### Development partners

All key informants reported that the development partners, especially WHO, UNICEF, and GAVI, play important roles in introducing a new vaccine in Bangladesh. The other development partners, such as World Bank, USAID, and UK-AID, are also the key actors in this regard. WHO and UNICEF provide technical support to Director, PHC and Programme Manager, EPI, about initiation and preparation of GAVI application, capacity assessment of cold-chains, and implementation of a new vaccine. Some key informants stated that the role of WHO and UNICEF is more important than the Government in terms of the introduction of new vaccines. They said that no matter whether the Government is interested or not but if WHO desires, they can introduce a vaccine in Bangladesh

In response to a question about the role of GAVI, the respondents mentioned that the GAVI mainly provides financial support for the introduction of new vaccines. They also added that the GAVI has some priority vaccines to introduce. If their requirements are fulfilled, they support us and provide vaccines.

#### ICC, NCIP, and Technical Sub-committees

The GAVI funds are used under the guidance of ICC. The ICC consists of members from different governments and NGO stakeholders. Secretary, MoHFW, is the chairperson of the committee. Other members include: Director General of DGHS; Director General of Family Planning (DGFP); Joint Secretary (PHC and WHO); Programme Manager, Child Health and Limited Curative Care; and Deputy Secretary of the MoHFW. The members of the ICC from other sectors include: Joint Secretary, Local Government Division, MoLGRD; Joint Secretary, Finance Division, Ministry of Finance; Director (Technical) of the Ministry of Environment; Secretary, National Polio Plus Committee of Rotary International; development partners, such as WHO, UNICEF, USAID, World Bank, UK-AID, JICA, Royal Netherlands, and Sida; representatives from NGOs; and others, such as icddr,b, Save the Children-USA, Immunization Consultant, and GAVI. ICC is part of the decision-making body in the introduction of new vaccines.

In Bangladesh, the NCIP formulates policy and monitors the EPI. It is the final body for taking steps in the introduction of new vaccines. The committee is chaired by the Secretary of MoHFW. Other members of the committee include: Director General, DGHS; Director General, DGFP; Joint Secretary (Public Health and WHO); Joint Chief (Planning); and Directors and Programme Managers, EPI, MoHFW. Other members also include professors at different medical colleges, representatives from professional associations, and development partners. The committee plays a vital role in making decisions on the introduction of new vaccines.

Under the supervision of the NCIP, the STSC has been formed to review the policy of EPI. Director General, DGHS, is the chair of the committee. Other members of the committee include: Directors of National Institute of Preventive and Social Medicine (NIPSOM), Institute of Epidemiology, Disease Control and Research (IEDCR), PHC, Maternal and Child Health, MoHFW; Professors at medical colleges; Programme Manager, EPI; representatives from professional associations; and representatives from WHO and UNICEF. The issue of introduction of new vaccines is first discussed in this committee for further processing.

### Decision-making process

Information on the burden of disease from research organizations leads to initiation of the process of introducing a new vaccine. Whenever information on the burden of disease comes from researchers and the Government and other technical personnel become convinced, the Government starts the process of introducing a new vaccine. The first step in the process of introducing a new vaccine is to discuss it in the STSC. The STSC also discusses about the possible funding sources, GAVI support, cold-chain capacity, and sources of other support in introducing the vaccine. If the STSC is satisfied and approves, a proposal is sent to the NCIP for its approval. Whenever the approval is provided from the NCIP, steps are taken to apply for GAVI support for the introduction of the vaccine. Before submitting the GAVI application, the ICC must endorse the proposal (Figure).

The respondents informed that whenever the NCIP and ICC approve the introduction of a new vaccine, the EPI headquarter starts the preparation of GAVI application for support. After receiving the complete application, the GAVI reviews it and sends it back with their comments and queries for clarification. After having satisfactory responses to their comments, the GAVI approves the application for the introduction of a new vaccine (Figure).

**Figure.**
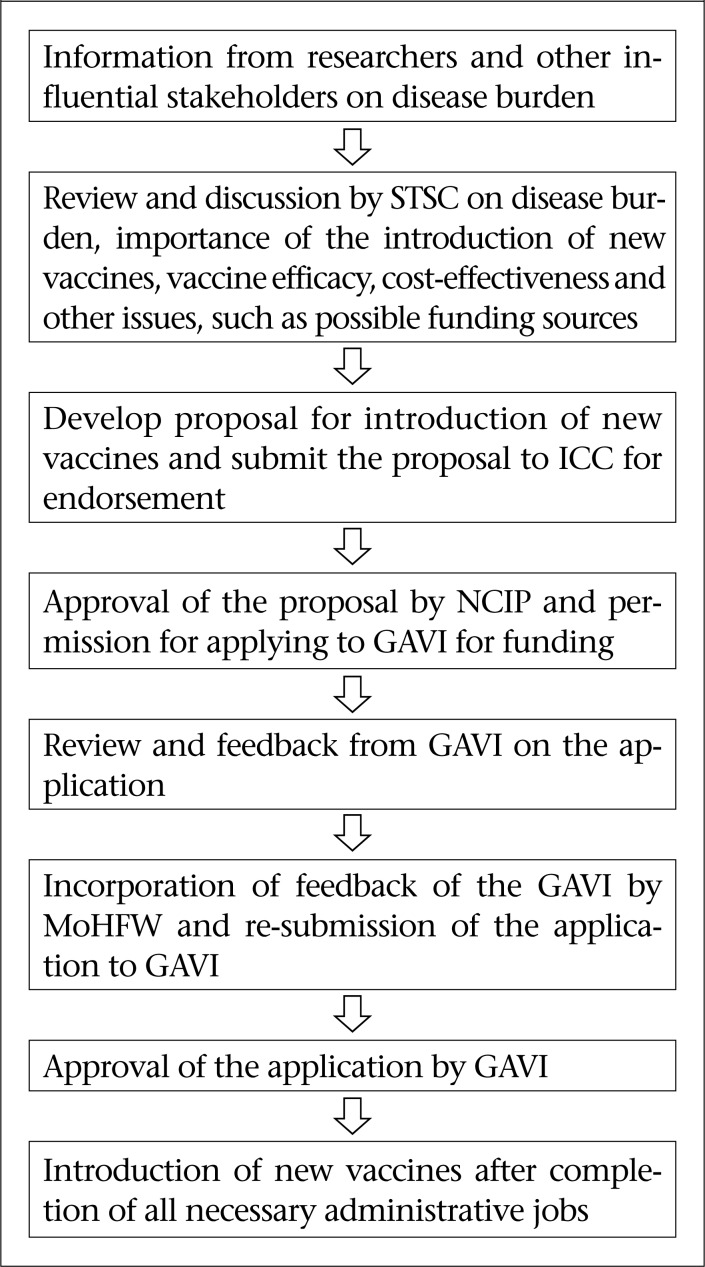
Decision-making process to adopt new vaccines in Bangladesh

The respondents actively involved in the preparation and submission of the GAVI application informed that almost two years are normally required to start the implementation of a vaccine after the submission of an application to the GAVI. The GAVI generally sends their comments six months after receipt of an application. It also takes some time to address the comments provided by the GAVI and resend the application to them. After resubmission of the application, it requires again around six months for the GAVI to take a decision and inform of their approval. According to the respondents, almost another one year is required to complete all the formalities and administrative work to start the implementation of the vaccine. The respondents from the EPI informed that they submitted an application to the GAVI for its support for the introduction of the Hib vaccine in 2007 in Bangladesh, which was finally implemented in 2009.

### Influencing factors in the introduction of new vaccines

The prevalence and burden of disease, findings of research on vaccine-preventable diseases, active participation of all key stakeholders, political issues relating to outbreaks of certain diseases, initiatives from international and local stakeholders, pressure from development partners, financial matters, and the GAVI support are the key factors that influence the introduction of new vaccines in Bangladesh. All the key informants stated that the GAVI, WHO, UNICEF, and other development partners have influenced the introduction of new vaccines. These international stakeholders not only influence by financing the introduction of new vaccines, they also provide technical support to the Government for the introduction.

The political issues relating to the outbreaks of certain diseases also influence decision-making. Some key informants stated that, in 2009, the newly-formed political government felt certain pressure of the Haemagglutinin type 1 and Neuraminidase type 1 (H1N1) pandemic in Bangladesh. At that time, the H1N1 pandemic became a political issue through the media coverage. Without any analysis of mortality and morbidity due to H1N1, the Government had to take decision to bring the H1N1 vaccine in the country to reduce the public panic prevalent at that time.

Most respondents stated that the financial factor is important for introducing new vaccines because new vaccines are usually more costly than the traditional vaccines. For introducing any new vaccine, the Government needs support from the development partners, particularly from the GAVI. The respondents also acknowledged the GAVI's contribution to two vaccines previously introduced, such as Hep-B and Hib. They stated that the Government can co-finance in introducing the new vaccines.

## DISCUSSION

In Bangladesh, while introducing new vaccines, decisions are jointly taken by the Government, academicians, researchers, representatives from different professional groups, and development partners. The Government, however, plays the key role. Without the contributions of other groups mentioned above, new vaccines might not be introduced in the country. The MoHFW initiates and forwards the GAVI application for a new vaccine after being vetted by the expert academicians. The researchers help the Government by providing relevant information to the policy-makers on the burden of disease, gathered from different studies and demand for new vaccines. The professional groups contribute to providing expert opinions about the introduction of new vaccines. The development partners, especially WHO and UNICEF, provide technical support to the concerned department of the MoHFW in the initiation and preparation of GAVI application, assessment of cold-chain capacity, and implementation of a new vaccine. The study participants mentioned that the role of WHO and UNICEF is remarkable in the introduction of new vaccines. All the actors work with positive attitudes toward the introduction of new vaccines. No negative attitude was observed among any actor involved in the introduction process of a new vaccine.

The process of introducing a new vaccine contributed to establishing a formal procedure in Bangladesh. Whenever information on the burden of disease becomes available from researchers and the Government and other technical personnel are convinced, the Government starts the process of introducing a new vaccine. The first step in the process is to discuss it in the STSC. The STSC also discusses about the possible funding sources, GAVI support, cold-chain capacity, and support from other sources in introducing the vaccine. If the Sub-committee is satisfied and approves, a proposal is sent to the NCIP for its approval. When the approval of the NCIP is accorded, steps are taken to apply for GAVI support for the introduction of the vaccine. Before submitting the GAVI application, the proposal has to be endorsed by the ICC. This formal procedure became structured and functional while introducing the Hib vaccine in Bangladesh in 2009.

The findings of the present study have shown that about two years are generally required to introduce a new vaccine after the submission of an application to the GAVI. The GAVI generally takes six months to send their comments to the Government. A considerable time is also needed in the resubmission of the application and making a decision by the GAVI. The findings of the study revealed that, after according approval by the GAVI, one year was required to complete all formalities and administrative work to start the implementation of the Hib vaccine. The slow process in the introduction and uptake of new vaccines is, thus, a concern in Bangladesh.

The prevalence and burden of a disease, research findings on vaccine-preventable diseases, political issues relating to outbreaks of certain diseases, initiatives from international and local stakeholders, pressure from the development partners, and financial matters are the key factors in the introduction of new vaccines in Bangladesh. The GAVI, WHO, UNICEF, and other development partners influence the decision in the introduction of a new vaccine. Political issues relating to the outbreaks of vaccine-preventable diseases also influence in making decisions. The Government needs financial support from the development partners, particularly from the GAVI, for introducing a new vaccine,. The issue of co-financing helped the Government take over the vaccine cost gradually. Therefore, the system of co-financing may be considered while introducing new vaccines in the country.

The GAVI Alliance's initiative to introduce new vaccines in Bangladesh is of utmost importance for children of the country. The role of Alliance in providing financial and technical resources for this purpose is crucial, especially given the high costs of such vaccines (17).

The findings of the study indicate that slashing the funding gaps for immunization and achieving financial sustainability will require several important actions in Bangladesh. For instance, a larger public-sector budget resulting from economic growth, greater government commitments to immunization within health budgets, greater multi-year commitments from donors, reduction in vaccine prices, and a major sustained effort by the GAVI Alliance to support Bangladesh to introduce new vaccines and to permit sufficient time and planning for a transition away from GAVI support may enable Bangladesh to become financially self-sustainable. Findings of others suggest similar actions for poor countries (17,18).

### Conclusions

The burden of disease, findings of research on vaccine-preventable diseases, political issues relating to outbreaks of certain diseases, initiatives of international and local stakeholders, pressure from the development partners, GAVI's support, and financial matters are the key factors in the introduction of new vaccines in Bangladesh. To expedite the introduction and uptake of new vaccines, it is important that the GAVI takes rapid action on the application for its support and the Government takes less time to complete the administrative work.

## ACKNOWLEDGEMENTS

The study was funded by the London School of Hygiene & Tropical Medicine (LSHTM), (Grant No. 803). icddr,b acknowledges with gratitude the commitment of LSHTM to its research efforts. The authors thank all key informants for their participation in the study. The authors also thank Mr. Wazed Ali, Senior Field Research Officer, icddr,b, for his assistance during data collection and writing of the manuscript.
